# Functional Magnetic Resonance Imaging-Based Analysis of Functional Connectivity in Chronic Stress: A Comparison of Stress-Induced and Recovery States

**DOI:** 10.3390/brainsci15101025

**Published:** 2025-09-23

**Authors:** Mi-Hyun Choi, Jaehui Kim

**Affiliations:** Department of Biomedical Engineering, Research Institute of Biomedical Engineering, School of ICT Convergence Engineering, College of Science & Technology, Konkuk University, 268 Chungwon-daero, Chungju-si 27478, Republic of Korea; jaehxx01@gmail.com

**Keywords:** chronic stress, functional connectivity, salience network, default mode network, frontoparietal network, fMRI

## Abstract

**Background/Objectives**: Chronic stress is associated with long-lasting alterations in brain function, particularly affecting the dynamic interactions between large-scale neural networks during stress and recovery. In this study, we compared changes in brain functional connectivity between states of stress induction and recovery in individuals with chronic stress and investigate the effects of chronic stress on functional brain networks. **Methods**: We used functional magnetic resonance imaging and ROI-to-ROI analysis to analyze functional connectivity in chronic stress (*n* = 36). The participants performed the Montreal Imaging Stress Task followed by a recovery phase. **Results**: The results showed that during the stress induction phase, connectivity between the salience and dorsal attention networks increased, demonstrating enhanced attention and emotional regulation. In contrast, during the recovery phase, connectivity between the default mode and the frontoparietal networks increased, demonstrating cognitive and emotional recovery after stress. Notably, we found that salience network activation continued during the recovery phase, suggesting that individuals with chronic stress may exhibit a continual state of alertness even after stress. **Conclusions**: Thus, our findings show that chronic stress can lead to the reconstruction of functional networks during the stress response and recovery, contributing to our understanding of the neurobiological correlates of stress-related impairment.

## 1. Introduction

Chronic stress is an increasing public health concern in modern society that has serious effects on both physical and mental health. In particular, chronic stress is a major risk factor for mental health disorders such as depression, anxiety, and posttraumatic stress disorder, with studies reporting changes in brain functional connectivity under these conditions [1,2].

Stress-related changes in functional brain networks such as the salience network (SN), default mode network (DMN), and frontoparietal network (FPN) have been reported in previous studies. For instance, Hermans et al. showed that the SN is activated in stressful situations and modulates emotional processing and attentional shifts [3], while van Marle et al. suggested that the DMN, involved in internal cognition, is suppressed during stress [4]. The FPN, responsible for cognitive control and decision-making, also shows changes in connectivity under stress conditions [5].

However, most previous studies have focused either on acute stress or on the structural effects of chronic stress, rather than on dynamic network responses during and after stress in individuals with chronic stress. For example, Dedovic et al. analyzed activation of the hypothalamic–pituitary–adrenal (HPA) axis in acute stress [6], and Liston et al. reported prefrontal structural changes under chronic stress [7].

Recent studies have increasingly explored both fMRI and EEG-based measures of functional connectivity in anxiety disorders and high trait anxiety [8,9]. Wang et al. [10] reported increased beta-band activity and decreased frontoparietal and parieto-temporal connectivity in patients with generalized anxiety disorder (GAD) via resting-state EEG. Langhammer et al. [11] observed altered resting-state functional connectivity between the insula, thalamus, and prefrontal cortex across different anxiety phenotypes using rs-fMRI. Additionally, dynamic EEG-fMRI mapping studies have begun to characterize how connectivity patterns fluctuate over time and in response to cognitive states [12]. These findings extend our understanding of connectivity disruptions beyond the acute stress context and underscore the need to examine recovery dynamics, particularly in chronic stress populations.

To date, few studies have examined how functional connectivity evolves from stress induction to recovery in people experiencing chronic stress. This represents a critical gap, especially considering that stress-related changes in the SN, DMN, and FPN may serve as potential biomarkers for early detection of mental health issues. Moreover, impairments such as PTSD and depression are known to originate from dysregulated stress responses and incomplete recovery.

Therefore, this study aimed to explore functional connectivity changes across brain networks during stress induction and the subsequent recovery phase in individuals with chronic stress. Understanding these dynamic processes may help identify neural patterns that distinguish maladaptive stress responses from adaptive regulation.

## 2. Materials and Methods

We used the Korean version of the Perceived Stress Scale (PSS) [13], a questionnaire used to measure stress levels, to identify individuals with chronic stress. Additionally, we used the PSS from previous studies [14,15] to categorize participants into acute stress and healthy groups. The PSS is a widely used instrument for assessing stress levels experienced by individuals. Many studies have used the PSS to diagnose stress, and the results of these studies have shown that it is a reliable instrument for assessing stress, with high internal consistency and test–retest reliability [13,16]. One study that analyzed the internal consistency of the PSS reported a Cronbach’s alpha of 0.84, demonstrating that the scale items measured the same concept (perceived stress) [13]. In another study, the test–retest reliability of the PSS was reported to be 0.85, which suggests that the scale is stable when assessing stress levels at an interval of two weeks [16]. The total PSS score ranges from 0 to 40 points, with higher scores indicating higher perceived stress levels. In general, a PSS score of 19 or higher indicates that the individual is under severe stress and may have difficulties in daily life. Therefore, this study focused on the functional connectivity changes between the stress-induced and recovery phases in participants with a PSS score of 19 or higher. Rather than comparing between groups, this study aimed to explore dynamic neural adaptation over time within the same individual.

### 2.1. Participants

Participants were recruited from healthy right-handed adults aged between 20 and 39 years who had no history of psychiatric or neurological disorders and were not taking any psychotropic medications. Prior to the experiment, all participants completed the Perceived Stress Scale (PSS), and only those with a score of 19 or higher were included in the study. Individuals with scores below this threshold were excluded.

After screening using the PSS, 36 individuals (27.8 ± 2.6 years) with a score ≥19 points were included in this study. We excluded individuals with conditions that could affect magnetic resonance imaging (MRI), including claustrophobia or metal objects in the body such as pacemakers or surgical wires. Before the experiment, we thoroughly explained the aims and protocol of the study to the participants and instructed them to limit external factors that could affect brain activity, such as smoking, alcohol consumption, and coffee consumption. The study was approved by the Institutional Review Committee of Konkuk University (IRB number: 7001355-202010-HR-405, approval date: 16 October 2022) and conforms to the provisions of the Declaration of Helsinki.

### 2.2. Selection of Stress-Induced Task

The Montreal Imaging Stress Task (MIST) was used to present a momentary stress task to the chronic stress group. The MIST was developed by Dedovic et al. [17] to induce psychosocial stress. In this task, the investigator provides negative feedback regarding the task performance to expose the participants to a situation in which they continually receive negative feedback from another person in an uncontrollable situation. Specifically, irrespective of their actual task performance, participants were informed that their performance was much worse than the virtual average performance, inducing social stress. This task is universally used to induce stress [14,15].

### 2.3. Experimental Design

In the rest phase, the participants rested in a comfortable state, while in the control phase, they solved elementary arithmetic problems without stress-inducing conditions. Conversely, in the stress task phase, the participants solved elementary arithmetic problems under stress-inducing conditions. The participants were visually presented with a time limit (3 s) in the form of a timer to induce stress during the stress task phase. Following each attempted answer, participants were shown their average score along with feedback on whether their response was correct, incorrect, or omitted, for a duration of 2 s before moving on to the subsequent item. During the recovery stage, they were instructed to relax comfortably after finishing the stress-inducing task. Prior to beginning the experiment, participants were encouraged to sustain an average score of 95 points.

### 2.4. Functional MRI Acquisition

Functional MRI data were acquired using a Siemens 3T system (Erlangen, Germany) equipped with a standard 16-channel head coil. A single-shot echo-planar imaging (EPI) sequence was applied to collect functional images across 29 contiguous slices aligned with the anterior–posterior commissure (AC–PC) plane. The acquisition parameters were: TR/TE = 2000/20 ms, field of view (FOV) = 240 mm, flip angle = 77°, matrix size = 128 × 128, slice thickness = 3 mm, and isotropic voxel size = 3.0 mm^3^. Structural images were collected using a T1-weighted 3D MPRAGE protocol with the following parameters: TR = 1900 ms, TE = 2.52 ms, FOV = 256 mm, flip angle = 9°, matrix = 256 × 256, slice thickness = 1 mm, resulting in voxel dimensions of 1.0 × 1.0 × 1.0 mm.

### 2.5. Functional Brain Imaging Analysis

Functional MRI data processing was conducted using Statistical Parametric Mapping (SPM12) software (Wellcome Department of Cognitive Neurology, London, UK; https://www.fil.ion.ucl.ac.uk/spm/software/spm12/; 15 January 2025).

Functional images were spatially aligned with the corresponding structural images via affine transformation included in SPM12. Within-subject time series were motion-corrected using a rigid-body six-parameter transformation, minimizing residuals through a least-squares optimization. The initial volume in each series served as the reference image for realignment. The corrected functional volumes were subsequently coregistered to the T1-weighted anatomical scan, followed by spatial normalization to the standard Montreal Neurological Institute (MNI) template. Inter-slice motion artifacts were mitigated through sinc interpolation. Additionally, high-pass temporal filtering (cut-off at 240 s) was applied to reduce low-frequency physiological noise, including respiration and cardiac fluctuations. Coregistered T1- and T2-weighted anatomical images were utilized in a multichannel segmentation procedure to generate probabilistic tissue maps encompassing six classifications: gray matter, white matter, cerebrospinal fluid, bone, soft tissue, and noise components. Prior to group-level statistical modeling, spatial smoothing was applied to the functional data using an isotropic Gaussian kernel with 8 mm full-width at half-maximum. Subsequent statistical analyses were carried out using the general linear model framework and Gaussian random field theory as implemented in SPM12.

Functional connectivity analysis was performed using the CONN toolbox (https://web.conn-toolbox.org/, 15 January 2025) integrated with SPM12. A region-to-region (ROI-to-ROI) connectivity approach was applied to identify connections associated with individual performance differences. A total of 105 ROIs were predefined based on the Harvard–Oxford cortical and subcortical structural atlases (https://neurovault.org/collections/262/, 15 January 2025), with the brainstem and cerebellum excluded from the analysis. Statistically significant ROI-to-ROI connectivity patterns were identified through cluster-level inference, employing spatial pairwise clustering (SPC) statistics with default CONN parameters, particularly in relation to recognition test outcomes. The strength of functional connectivity between ROIs is defined as the temporal correlation of BOLD signals between each region, reflecting the degree of synchronized neural activity across distinct brain areas.

T-statistics across the full ROI-to-ROI connectivity matrix were computed using a general linear model (GLM). A total of 11 regions of interest (ROIs) were selected as seeds, distributed across four core brain networks. The default mode network (DMN) consisted of the medial prefrontal cortex (MPFC), lateral parietal lobe (LP), and posterior cingulate cortex (PCC). The salience network (SN) included the anterior cingulate cortex (ACC), anterior insula (A. Insula), rostral prefrontal cortex (RPFC), and supramarginal gyrus (SMG). The dorsal attention network (DAN) incorporated the frontal eye fields (FEF) and intraparietal sulcus (IPS). Lastly, the frontoparietal network (FPN) was defined by the lateral prefrontal cortex (LPFC) and posterior cingulate cortex (PCC).

### 2.6. Statistical Analysis

To correct for the issue of multiple comparisons in the ROI-to-ROI connectivity analysis, we applied the seed-level False Discovery Rate (FDR) correction method provided by the CONN toolbox, with a significance threshold set at *p* < 0.05. This approach effectively controls the Type I error rate that can occur across a large number of ROI-to-ROI tests, while maintaining statistical power. Initially, we set a voxel-level threshold of *p* < 0.01 (uncorrected), and only interpreted cluster-level results that survived FDR correction (*p* < 0.05, FDR corrected).

## 3. Results

**Figure 1:**
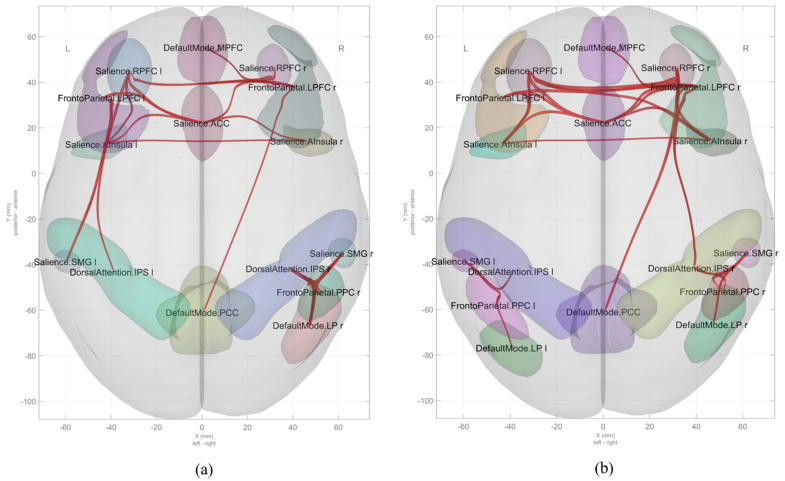
Functional brain connectivity patterns during stress induction (**a**) and recovery (**b**) phases in the chronic stress group (*n* = 36). The figure displays significant increases in connectivity between key brain networks, including the Salience Network (SN), Frontoparietal Network (FPN), Default Mode Network (DMN), and Dorsal Attention Network (DAN). Notably, persistent SN activation is observed in the recovery phase, while increased connectivity between DMN and FPN emerges post-stress.

**Figure 2:**
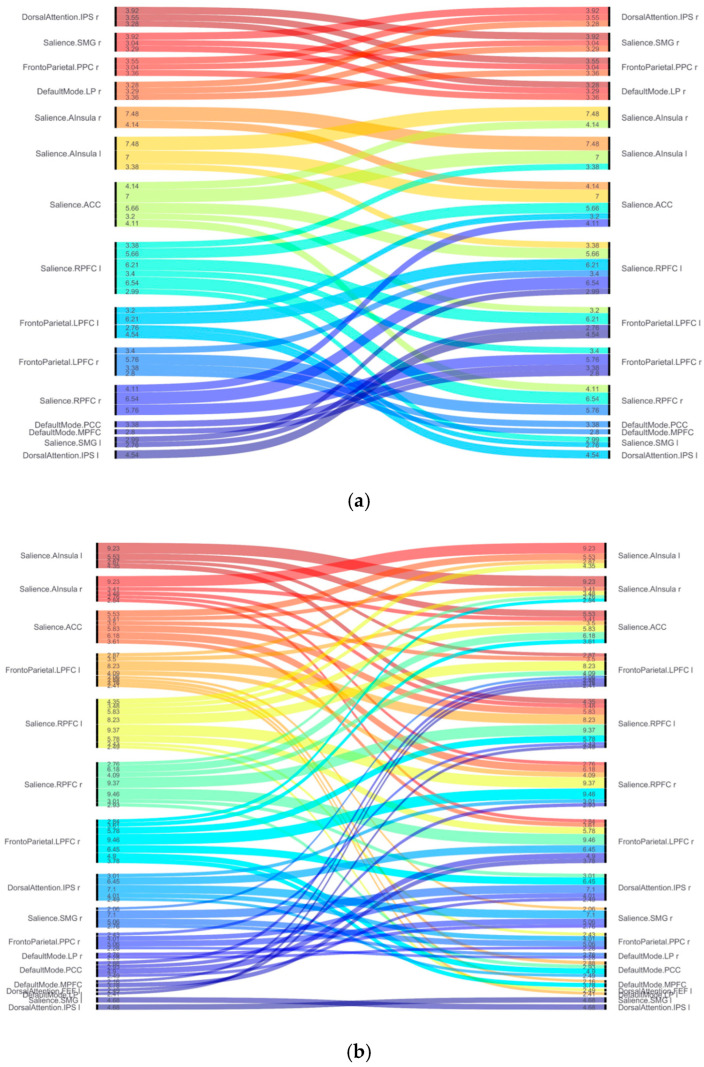
ROI-to-ROI functional connectivity during the stress and recovery phases in individuals with chronic stress. (**a**) Sankey-style diagram visualizing functional connectivity during the stress-induction phase. Prominent connections are observed within the salience network (SN), as well as between the SN, frontoparietal network (FPN), and dorsal attention network (DAN), reflecting heightened attention and emotional regulation under stress. (**b**) Sankey-style diagram illustrating connectivity during the recovery phase. Increased inter-network integration is observed, particularly between the SN, FPN, and default mode network (DMN), suggesting engagement of cognitive and emotional recovery mechanisms. Notably, the SN remains active in both phases, indicating potential sustained hypervigilance in individuals with chronic stress.

Table 1 and Table 2 and Figure 1 and Figure 2 show the results for functional brain networks under stress-induced and recovery conditions in the chronic stress group.

During the stress-induction phase (Table 1 and Figure 1a), a clear increase was observed in the activity of the salience network (SN). In particular, there was strong activation in the connections between the anterior insula (A Insula), rostral prefrontal cortex (RPFC), and anterior cingulate cortex (ACC). The connection between the right and left insula showed the strongest activation (T(35) = 7.48, *p* < 0.001), and the connectivity between the left insula and the ACC also increased significantly (T(35) = 7.00, *p* < 0.001). Additional connectivity was observed between the left and right RPFC (T(35) = 6.54, *p* < 0.001), and between the left rostral prefrontal cortex (RPFC) and the left lateral prefrontal cortex (LPFC; part of the frontoparietal network; T(35) = 6.21, *p* < 0.001). These results are visualized in Figure 2a, which depicts the stress-phase network as a Sankey diagram, highlighting the dominant connections within and between key networks.

In the recovery phase following the stress task (Table 2 and Figure 1b), the connectivity pattern shifted. While internal connectivity within the salience network remained elevated, inter-network connectivity—particularly between the salience network, frontoparietal network (FPN), and default mode network (DMN)—was markedly enhanced. The strongest increase in connectivity was observed between the right rostral prefrontal cortex (SN) and right left lateral prefrontal cortex (FPN; T(35) = 9.46, *p* < 0.001). High connectivity was also maintained between the left and right rostral prefrontal cortex (T(35) = 9.37, *p* < 0.001), and between the left and right A Insula (T(35) = 9.23, *p* < 0.001). Furthermore, functional connectivity between the right lateral prefrontal cortex (FPN) and the posterior cingulate cortex (PCC; DMN) increased significantly (T(35) = 4.90, *p* < 0.001), suggesting that the DMN began to recover its baseline function as the stress response subsided. These patterns are summarized in Figure 2b, which highlights the expanded and more integrated inter-network architecture in the recovery phase.

## 4. Discussion

The purpose of this study was to investigate the effects of chronic stress on functional brain connectivity during stress induction and subsequent recovery. Focusing on within-subject variation, we sought to identify dynamic neural adaptations associated with psychosocial stress in individuals with high perceived stress. Inter-ROI analysis visualized through tables and Sankey-style diagrams revealed distinct patterns of network engagement across the two phases.

### 4.1. Comparison of Network Analysis Results upon Stress Induction in a Group with Chronic Stress

This study revealed a clear increase in internal connectivity in the SN when individuals with chronic stress performed stress-inducing tasks. Specifically, stronger functional connectivity among the insula, RPFC, and ACC was observed. This finding indicated that stressful situations lead to prominent activation of the SN, which plays an important role in attention and emotion regulation.

Stress has also been reported to affect the functional connectivity in the brain. For example, Zhang et al. [18] reported that acute stress could alter connectivity in the SN and could be a potential indicator of trauma-related symptoms. In addition, Clemens et al. [19] discovered that functional connectivity in the SN increased after social stress, while FPN connectivity decreased. However, the findings are novel in that specific changes in the SN were identified during stress induction in individuals with chronic stress, including stronger functional connectivity between specific brain regions. Thus, these results provide a deeper understanding of how chronic stress affects patterns of activation in specific brain networks. Notably, decreased activity in the DMN was observed during stress induction, indicating that responses to external stimuli were prioritized over internal cognition or self-related processing in stressful situations. This decrease in DMN activity can also be interpreted as an adaptive mechanism to stress in the brain. Moreover, this is consistent with previous studies which have reported that stress affects DMN activity. For example, van Marle et al. (2010) reported that acute stress suppressed DMN activity and increased attention to external stimuli [4]. Thus, our results suggest that the brain’s resources are diverted towards processing external stimuli in stressful situations.

To summarize, stress induction in the chronic stress group resulted in activation of the SN and simultaneous suppression of the DMN, which reflects the dual response of the brain in stressful situations of attending to external stimuli while suppressing internal cognition. These findings provide important insights into the complex adaptive mechanisms of the brain in response to stress.

### 4.2. Comparison of Network Analysis Results in the Recovery Phase in a Group with Chronic Stress

Notably, the internal connectivity of the SN was maintained in the recovery phase after stress induction, and an increased interaction between the FPN and DMN was observed. In particular, functional connectivity was strengthened between the right RPFC (SN) and LPFC (FPN), suggesting that cooperation between networks related to cognitive control increases after stress. van Oort et al. [20] discovered that the functional connectivity of the SN increases after social stress, whereas FPN connectivity decreases. Additionally, Liu et al. [21] reported that the experience of COVID-19-related trauma could affect DMN functional connectivity and that psychological resilience could protect this functional connectivity. Thus, our results suggest that the DMN plays an important role in recovery from stress. Furthermore, Chan et al. [22] analyzed the effects of traumatic experiences on DMN function and reported that while DMN connectivity decreased in groups exposed to trauma, they recovered over time. The increase in connectivity between the DMN and FPN that observed during stress recovery in this study is consistent with these results and suggests that the functional connectivity of the DMN could gradually recover during recovery after stress induction.

However, previous studies have mostly analyzed network changes during acute stress; only a few studies have directly compared the patterns of increasing connectivity between the FPN and DMN during recovery. Notably, internal connectivity within the SN remained persistently high even after stress induction in the chronic stress group. This suggests that individuals who have experienced chronic stress may not recover completely after stress but may maintain persistently high alertness.

While this persistent salience network activation may suggest a state of hypervigilance, it is important to consider multiple interpretations. Such sustained activity could reflect an adaptive mechanism of maintaining heightened readiness in individuals under chronic stress. Conversely, it may indicate a maladaptive failure to return to baseline functioning after stress. Therefore, the prolonged SN activation observed during the recovery phase should be interpreted with caution, considering both its potential benefits and detrimental effects. Consequently, this finding implies that changes in neural networks in people with chronic stress could differ from the normal acute stress response and could have long-term neurological effects. Therefore, future studies should precisely analyze the role of the DMN in the process of stress recovery in chronic stress groups and review the effects of psychological resilience and emotional regulation strategies on the recovery of functional connectivity.

### 4.3. Comparison of Network Changes Between Stress Induction and Recovery in Chronic Stress Group

A characteristic increase in the connectivity among the FPN, DAN, DMN, and SN was observed during stress induction. Specifically, increased connectivity was observed Frontoparietal PPC (R) and DefaultMode.LP (R), DorsalAttention.IPS (R) and DefaultMode.LP (R), and FrontoParietal.LPFC (L) and DorsalAttention.IPS (L). These changes imply that attention and cognitive regulation are enhanced in stressful situations [18]. Additionally, within the SN, Salience.RPFC (L)-Salience.SMG (L) and Frontoparietal.LPFC (L)-Salience.SMG (L) connectivity increased, demonstrating the activation of neural networks related to emotional regulation and the allocation of attentional resources when stress is induced [2].

In contrast, the recovery phase was observed patterns of increased connectivity between the FPN, DAN, and SN. Specifically, increases in FrontoParietal.LPFC (R) and DorsalAttention.IPS (R), FrontoParietal.LPFC (L) and Salience.RPFC (R), and Salience.ACC and FrontoParietal.LPFC (R) connectivity were observed. This may reflect the gradual recovery of cognitive and emotional control after stress induction [21]. Additionally, increases in Salience.AInsula (R)-Salience.RPFC (L), Salience.RPFC (R)-DorsalAttention.IPS (R), and Salience.AInsula (L)-Frontoparietal. LPFC (L) connectivity were observed, indicating the activation of functions related to autonomous regulation and emotional control during recovery [22].

Collectively, these results suggest that different neural networks play prominent roles in the stress induction and recovery phases. While functional connectivity increases in the FPN, DAN, and SN, which are responsible for attention and cognitive regulation during stress induction, the recovery phase is characterized by interactions between the SN and FPN, which are related to emotional and autonomic regulation [20]. During recovery, a marked increase was observed in connectivity between the insula and RPFC within the SN, which could signify the activation of emotional regulation and self-control processes after stress [23,24].

Thus, our findings are significant because they demonstrate that brain networks are reconstructed differently during stress response and recovery. Moreover, persistent activation of the SN was observed during the recovery phase in the chronic stress group, suggesting that these individuals might continuously maintain a state of alertness [18].

This study has several limitations. First, it was conducted on a relatively small sample of young adults (*n* = 36), which limits the generalizability of the findings to other age groups or clinical populations. Second, the study focused solely on a chronic stress group and did not include a healthy control group. Future studies should include comparative analysis with controls to better understand the specific effects of chronic stress on functional connectivity. Third, the analysis relied exclusively on fMRI-based neural indicators without incorporating behavioral or physiological measures such as cortisol, heart rate variability, or subjective stress ratings. Fourth, this study focused only on static functional connectivity and did not explore dynamic changes in network interactions over time.

This study compared changes in functional connectivity between the stress induction and recovery phases in individuals with chronic stress. The results provide foundational insights into the neural adaptation mechanisms associated with chronic stress and suggest the potential of functional connectivity changes as biomarkers for the early detection of psychiatric disorders. Notably, the sustained connectivity observed in specific networks (e.g., the salience network) even during the recovery phase may reflect reduced stress resilience or persistent hypervigilance. These findings could be applied to mental health assessment and the development of intervention strategies.

## 5. Conclusions

This study compared functional connectivity between the stress induction and recovery phases in individuals with chronic stress. During the stress phase, connectivity increased in the salience, dorsal attention, and frontoparietal networks. In the recovery phase, connectivity between the default mode network and the frontoparietal network increased. Notably, the salience network maintained high connectivity even during recovery, suggesting persistent hypervigilance following stress exposure in chronic stress individuals. These findings highlight the potential long-term impact of chronic stress on the reconfiguration of brain networks.

## Figures and Tables

**Table 1:** Significant ROI-to-ROI functional connectivity during the stress induction phase in the chronic stress group (*n* = 36). The analysis was conducted using the CONN toolbox, with thresholding at *p* < 0.01 (uncorrected) and cluster-level FDR correction at *p* < 0.05. Bolded connections indicate uniquely increased connectivity observed only during the stress phase. (R: Right/L: Left), Values in parentheses (x, y, z) represent MNI space coordinates.

Analysis Unit	Statistic		
Cluster 1/11 Mass = 571.34		*p*-FDR	*p*-FWE
Salience.AInsula (R) (47,14,0)-Salience.AInsula (L) (−44,13,1)	T(35) = 7.48	0.000000	0.000002
Salience.AInsula (L) (−44,13,1)-Salience.ACC (0,22,35)	T(35) = 7.00	0.000000	0.000003
Salience.RPFC (L) (−32,45,27)-Salience.RPFC (R) (32,46,27)	T(35) = 6.54	0.000000	0.000009
Salience.RPFC (L) (−32,45,27)-FrontoParietal.LPFC (L) (−43,33,28)	T(35) = 6.21	0.000000	0.000017
Salience.ACC (0,22,35)-Salience.RPFC (L) (−32,45,27)	T(35) = 5.66	0.000002	0.000061
Salience.AInsula (R) (47,14,0)-Salience.ACC (0,22,35)	T(35) = 4.14	0.000211	0.003960
Salience.ACC (0,22,35)-Salience.RPFC (R) (32,46,27)	T(35) = 4.11	0.000224	0.003960
Salience.RPFC (L) (−32,45,27)-FrontoParietal.LPFC (R) (41,38,30)	T(35) = 3.40	0.001714	0.016891
Salience.AInsula (L) (−44,13,1)-Salience.RPFC (L) (−32,45,27)	T(35) = 3.38	0.001816	0.016891
Salience.ACC (0,22,35)-FrontoParietal.LPFC (L) (−43,33,28)	T(35) = 3.20	0.002934	0.021810
Cluster 2/11 Mass = 140.14			
DorsalAttention.IPS (R) (39,−42,54)-Salience.SMG (R) (62,−35,32)	T(35) = 3.92	0.000395	0.005573
DorsalAttention.IPS (R) (39,−42,54)-FrontoParietal.PPC (R) (52,−52,45)	T(35) = 3.55	0.001117	0.012729
FrontoParietal.PPC (R) (52,−52,45)-DefaultMode.LP (R) (47,−67,29)	T(35) = 3.36	0.001877	0.016891
Salience.SMG (R) (62,−35,32)-DefaultMode.LP (R) (47,−67,29)	T(35) = 3.29	0.002290	0.018904
DorsalAttention.IPS (R) (39,−42,54)-DefaultMode.LP (R) (47,−67,29)	T(35) = 3.28	0.002375	0.018904
Salience.SMG (R) (62,−35,32)-FrontoParietal.PPC (R) (52,−52,45)	T(35) = 3.04	0.004488	0.030700
Cluster 3/11 Mass = 104.81			
FrontoParietal.LPFC (R) (41,38,30)-Salience.RPFC (R) (32,46,27)	T(35) = 5.76	0.000002	0.000056
FrontoParietal.LPFC (R) (41,38,30)-DefaultMode.PCC (1,−61,38)	T(35) = 3.38	0.001790	0.016891
FrontoParietal.LPFC (R) (41,38,30)-DefaultMode.MPFC (1,55,−3)	T(35) = 2.80	0.008196	0.051479
Cluster 4/11 Mass = 74.35			
FrontoParietal.LPFC (L) (−43,33,28)-DorsalAttention.IPS (L) (−39,−43,52)	T(35) = 4.54	0.000064	0.001561
Salience.RPFC (L) (−32,45,27)-Salience.SMG (L) (−60,−39,31)	T(35) = 2.99	0.005043	0.033165
FrontoParietal.LPFC (L) (−43,33,28)-Salience.SMG (L) (−60,−39,31)	T(35) = 2.76	0.009178	0.054120

**Table 2:** Significant ROI-to-ROI functional connectivity during the recovery phase following stress induction in the chronic stress group. (R: Right/L: Left), Values in parentheses (x, y, z) represent MNI space coordinates.

Analysis Unit	Statistic		
Cluster 1/5 Mass = 1450.45		*p*-FDR	*p*-FWE
Salience.RPFC (R) (32,46,27)-FrontoParietal.LPFC (R) (41,38,30)	T(35) = 9.46	0	0
Salience.RPFC (L) (−32,45,27)-Salience.RPFC (R) (32,46,27)	T(35) = 9.37	0	0
Salience.AInsula (L) (−44,13,1)-Salience.AInsula (R) (47,14,0)	T(35) = 9.23	0	0
FrontoParietal.LPFC (L) (−43,33,28)-Salience.RPFC (L) (−32,45,27)	T(35) = 8.23	0	0
DorsalAttention.IPS (R) (39,−42,54)-Salience.SMG (R) (62,−35,32)	T(35) = 7.10	0	0.000001
FrontoParietal.LPFC (R) (41,38,30)-DorsalAttention.IPS (R) (39,−42,54)	T(35) = 6.45	0	0.000006
Salience.ACC (0,22,35)-Salience.RPFC (R) (32,46,27)	T(35) = 6.18	0	0.000011
Salience.ACC (0,22,35)-Salience.RPFC (L) (−32,45,27)	T(35) = 5.83	0.000001	0.000027
Salience.RPFC (L) (−32,45,27)-FrontoParietal.LPFC (R) (41,38,30)	T(35) = 5.78	0.000002	0.000029
Salience.AInsula (L) (−44,13,1)-Salience.ACC (0,22,35)	T(35) = 5.53	0.000003	0.000054
Salience.SMG (R) (62,−35,32)-FrontoParietal.PPC (R) (52,−52,45)	T(35) = 5.06	0.000013	0.000208
Salience.AInsula (L) (−44,13,1)-Salience.RPFC (L) (−32,45,27)	T(35) = 4.35	0.000113	0.001377
FrontoParietal.LPFC (L) (−43,33,28)-Salience.RPFC (R) (32,46,27)	T(35) = 4.09	0.000244	0.002778
DorsalAttention.IPS (R) (39,−42,54)-FrontoParietal.PPC (R) (52,−52,45)	T(35) = 4.01	0.000302	0.003224
Salience.ACC (0,22,35)-FrontoParietal.LPFC (R) (41,38,30)	T(35) = 3.61	0.000937	0.008897
Salience.ACC (0,22,35)-FrontoParietal.LPFC (L) (−43,33,28)	T(35) = 3.50	0.001291	0.01162
Salience.AInsula (R) (47,14,0)-Salience.RPFC (L) (−32,45,27)	T(35) = 3.48	0.001361	0.011635
Salience.AInsula (R) (47,14,0)-Salience.ACC (0,22,35)	T(35) = 3.41	0.00163	0.01327
Salience.RPFC (R) (32,46,27)-DorsalAttention.IPS (R) (39,−42,54)	T(35) = 3.01	0.004835	0.035948
Salience.AInsula (L) (−44,13,1)-FrontoParietal.LPFC (L) (−43,33,28)	T(35) = 20.87	0.00695	0.044014
Salience.AInsula (R) (47,14,0)-FrontoParietal.LPFC (R) (41,38,30)	T(35) = 2.84	0.007526	0.044377
Salience.AInsula (R) (47,14,0)-Salience.RPFC (R) (32,46,27)	T(35) = 2.76	0.00906	0.050975
Salience.SMG (R) (62,−35,32)-DefaultMode.LP (R) (47,−67,29)	T(35) = 2.76	0.009241	0.050975
Cluster 2/5 Mass = 93.77			
FrontoParietal.LPFC (R) (41,38,30)-DefaultMode.PCC (1,−61,38)	T(35) = 4.90	0.000022	0.00031
FrontoParietal.LPFC (R) (41,38,30)-DefaultMode.MPFC (1,55,−3)	T(35) = 3.78	0.000585	0.005889
Salience.RPFC (R) (32,46,27)-DefaultMode.PCC (1,−61,38)	T(35) = 2.93	0.005908	0.04182
Cluster 3/5 Mass = 76.97			
Salience.SMG (L) (−60,−39,31)-DorsalAttention.IPS (L) (−39,−43,52)	T(35) = 4.68	0.000042	0.000557
DefaultMode.LP (L) (−39,−77,33)-FrontoParietal.PPC (L) (−46,−58,49)	T(35) = 20.92	0.006114	0.04182
FrontoParietal.PPC (L) (−46,−58,49)-Salience.SMG (L) (−60,−39,31)	T(35) = 2.84	0.007384	0.044377

## Data Availability

The data presented in this study are available on request from the corresponding author due to Privacy Protection and Research Ethics.

## References

[1] Buckner R.L., Andrews-Hanna J.R., Schacter D.L. (2008). The Brain’s Default Network: Anatomy, Function, and Relevance to Disease. Ann. N. Y. Acad. Sci..

[2] Menon V. (2011). Large-Scale Brain Networks and Psychopathology: A Unifying Triple Network Model. Trends Cogn. Sci..

[3] Hermans E.J., van Marle H.J.F., Ossewaarde L., Henckens M.J.A.G., Qin S., van Kesteren M.T.R., Schoots V.C., Cousijn H., Rijpkema M., Oostenveld R. (2011). Stress-Related Noradrenergic Activity Prompts Large-Scale Neural Network Reconfiguration. Science.

[4] van Marle H.J.F., Hermans E.J., Qin S., Fernández G. (2010). Enhanced Resting-State Connectivity of Amygdala in the Immediate Aftermath of Acute Psychological Stress. NeuroImage.

[5] Segal A., Charquero-Ballester M., Vaisvasser S., Cabral J., Ben-Zion Z., Vidaurre D., Stark E., McManners H., Woolrich M., Ehlers A. (2025). Brain network dynamics following induced acute stress: A neural marker of psychological vulnerability to real-life chronic stress. Psychol. Med..

[6] Dedovic K., D’Aguiar C., Pruessner J.C. (2009). What Stress Does to Your Brain: A Review of Neuroimaging Studies. Can. J. Psychiatry.

[7] Liston C., McEwen B.S., Casey B.J. (2009). Psychosocial Stress Reversibly Disrupts Prefrontal Processing and Attentional Control. Proc. Natl. Acad. Sci. USA.

[8] Afek N., Harmatiuk D., Gawłowska M., Ferreira J.M.A., Golonka K., Tukaiev S., Popov A., Marek T. (2025). Functional connectivity in burnout syndrome: A resting-state EEG study. Front. Hum. Neurosci..

[9] Lupinsky D., Nasseef M.T., Parent C., Craig K., Diorio J., Zhang T.Y., Meaney M.J. (2025). Resting-state fMRI reveals altered functional connectivity associated with resilience and susceptibility to chronic social defeat stress in mouse brain. Mol. Psychiatry.

[10] Wang H., Mou S., Pei X., Zhang X., Shen S., Zhang J., Shen X., Shen Z. (2025). The power spectrum and functional connectivity characteristics of resting-state EEG in patients with generalized anxiety disorder. Sci. Rep..

[11] Langhammer T., Hilbert K., Adolph D., Arolt V., Bischoff S., Böhnlein J., Cwik J.C., Dannlowski U., Deckert J., Domschke K. (2025). Resting-state functional connectivity in anxiety disorders: A multicenter fMRI study. Mol. Psychiatry.

[12] Chu L.H., Chau C.Q., Kamel N., Thanh H.H.T., Yahya N. (2024). Functional excitation-inhibition ratio for social anxiety analysis and severity assessment. Front. Psychiatry.

[13] Cohen S., Janicki-Deverts D. (2012). Who’s Stressed? Distributions of Psychological Stress in the United States in Probability Samples from 1983, 2006, and 2009. J. Appl. Soc. Psychol..

[14] Choi M.-H. (2023). A Pilot Study: Extraction of a Neural Network and Feature Extraction of Generation and Reduction Mechanisms Due to Acute Stress. Brain Sci..

[15] Choi M.-H., Choi J.-S. (2024). Comparing Brain Activation Patterns in Stress-Induced and Post-Stress Recovery States of Highly and Moderately Stressed Individuals. Appl. Sci..

[16] Lee E.H. (2012). Review of the Psychometric Evidence of the Perceived Stress Scale. Asian Nurs. Res..

[17] Dedovic K., Renwick R., Mahani N.K., Engert V., Lupien S.J., Pruessner J.C. (2005). The Montreal Imaging Stress Task: Using Functional Imaging to Investigate the Effects of Perceiving and Processing Psychosocial Stress in the Human Brain. J. Psychiatry Neurosci..

[18] Zhang W., Kaldewaij R., Hashemi M.M., Koch S.B.J., Smit A., van Ast V.A., Beckmann C.F., Klumpers F., Roelofs K. (2022). Acute-Stress-Induced Change in Salience Network Coupling Prospectively Predicts Post-Trauma Symptom Development. Transl. Psychiatry.

[19] Clemens B., Wagels L., Bauchmüller M., Bergs R., Habel U., Kohn N. (2017). Alerted Default Mode: Functional Connectivity Changes in the Aftermath of Social Stress. Sci. Rep..

[20] Van Oort J., Tendolkar I., Hermans E.J., Mulders P.C., Beckmann C.F., Schene A.H., Fernández G., van Eijndhoven P.F. (2017). How the Brain Connects in Response to Acute Stress: A Review at the Human Brain Systems Level. Neurosci. Biobehav. Rev..

[21] Liu X., Zhao Y., Suo X., Zhang X., Pan N., Kemp G.J., Gong Q., Wang S. (2023). Psychological Resilience Mediates the Protective Role of Default-Mode Network Functional Connectivity Against COVID-19 Vicarious Traumatization. Transl. Psychiatry.

[22] Chan A., Harvey P., Hernandez-Cardenache R., Alperin N., Lee S., Hunt C., Petersen N., Northoff G., Robertson N., Ouyang J. (2024). Trauma and the Default Mode Network: Review and Exploratory Study. Front. Behav. Neurosci..

[23] Hermans E.J., Henckens M.J.A.G., Joëls M., Fernández G. (2014). Dynamic Adaptation of Large-Scale Brain Networks in Response to Acute Stressors. Trends Neurosci..

[24] Goldfarb E.V., Scheinost D., Fogelman N., Seo D., Sinha R. (2022). High-Risk Drinkers Engage Distinct Stress-Predictive Brain Networks. Biol. Psychiatry Cogn. Neurosci. Neuroimaging.

